# Deep Learning Methods for Interpretation of Pulmonary CT and X-ray Images in Patients with COVID-19-Related Lung Involvement: A Systematic Review

**DOI:** 10.3390/jcm12103446

**Published:** 2023-05-13

**Authors:** Min-Ho Lee, Adai Shomanov, Madina Kudaibergenova, Dmitriy Viderman

**Affiliations:** 1 School of Engineering and Digital Sciences, Nazarbayev University, Kabanbay Batyr Ave. 53, Astana 010000, Kazakhstan; 2 School of Medicine, Nazarbayev University, 5/1 Kerey and Zhanibek Khandar Str., Astana 010000, Kazakhstan

**Keywords:** artificial intelligence, deep learning, systematic review, X-ray, computerized tomography, COVID-19

## Abstract

SARS-CoV-2 is a novel virus that has been affecting the global population by spreading rapidly and causing severe complications, which require prompt and elaborate emergency treatment. Automatic tools to diagnose COVID-19 could potentially be an important and useful aid. Radiologists and clinicians could potentially rely on interpretable AI technologies to address the diagnosis and monitoring of COVID-19 patients. This paper aims to provide a comprehensive analysis of the state-of-the-art deep learning techniques for COVID-19 classification. The previous studies are methodically evaluated, and a summary of the proposed convolutional neural network (CNN)-based classification approaches is presented. The reviewed papers have presented a variety of CNN models and architectures that were developed to provide an accurate and quick automatic tool to diagnose the COVID-19 virus based on presented CT scan or X-ray images. In this systematic review, we focused on the critical components of the deep learning approach, such as network architecture, model complexity, parameter optimization, explainability, and dataset/code availability. The literature search yielded a large number of studies over the past period of the virus spread, and we summarized their past efforts. State-of-the-art CNN architectures, with their strengths and weaknesses, are discussed with respect to diverse technical and clinical evaluation metrics to safely implement current AI studies in medical practice.

## 1. Introduction

The novel coronavirus (SARS-CoV-2) emerged in early December 2019 and has since become a global pandemic threatening humanity [1]. COVID-19, the disease caused by this virus, presents with non-specific symptoms such as cough, fever, myalgia, headache, gastrointestinal dysfunction, and other flu-like symptoms, thereby making it challenging to differentiate from other viral upper respiratory diseases, especially in the early stages [2]. However, the timely diagnosis and management of affected patients could reduce mortality and the spread of COVID-19 [1,2].

The standard diagnostic tests used to confirm COVID-19 diagnosis are real-time RT-PCR and the reverse transcription polymerase chain reaction (RT-PCR) [3,4]. Chest-computed tomography (CCT) is a radiological diagnostic tool used to detect the pulmonary manifestations and complications of COVID-19, such as pneumonia and acute respiratory distress syndrome. The main radiological features of COVID-19 are asymmetric peripheral ground-glass opacities (GGOs) in the absence of pleural effusions [5]. However, given the high volume of cases that radiologists need to analyze, the manual interpretation of chest CT scans can be monotonous, tedious, and prone to diagnostic errors. This is where automated artificial intelligence tools can come in handy by facilitating and double-checking physicians’ tasks, as well as reducing the likelihood of diagnostic errors.

Recent advances in artificial intelligence, especially deep learning (DL) algorithms, have shown great potential in accurately interpreting medical images, including chest CT scans [6]. CNN, one of the DL algorithms, can learn the most important representations through layer-by-layer feature analysis [7] and has been successfully adapted to analyze chest CT images for COVID-19 detection [8,9].

Although the characteristics of the current CNN models have been widely tested and described in the literature, there is no systematic review and meta-analysis focusing on the application of CNN in the detection of pulmonary involvement in COVID-19 patients. Therefore, the aim of this systematic review is to study the performance, benefits, and risks of CNN for the detection of pulmonary manifestations in COVID-19 patients.

One of the major challenges in developing CNN models for COVID-19 detection is the limited size of medical datasets, especially in COVID-19 cases. To address this issue, advanced augmentation approaches based on conventional generative adversarial networks (GANs) have been successfully applied to acquire a sufficient number of clinical data samples. Recently, publicly available datasets have been created by many research foundations, and CNN architecture with data-invariant performance has become one of the most challenging issues in clinical CNN study.

Another challenge is constructing a parameter-invariant CNN framework that assures robust performance on the variations of medical imaging data. Techniques such as semi-automated image processing software or CNN-based segmentation have been developed to overcome this issue by extracting the region of interest. More recent end-to-end CNN architectures have shown comparable or even superior performance to conventional pipeline systems.

For the performance evaluation, we utilized popular CNN architectures designed primarily for the computer vision field as our baseline models. These models included VGG, Inception, DenseNet, GoogLeNet, ResNet, and more extensive versions of the individual networks. Diverse state-of-the-art CNN models have been proposed, which vary in terms of model structures such as activation functions, filter/kernel size, model parameters, and depth of the network. These models aim to improve the representational ability of feature maps to overcome known problems such as overfitting, vanishing gradient, computational cost, etc. Transfer learning, ensemble metal-classifiers, and fine-tuning based on pre-trained CNN models have been successfully implemented in clinical imaging data. However, the lack of a common framework, as well as a benchmark dataset, make it challenging to provide a reliable comparison of the state-of-art methodologies.

Meanwhile, ensuring the reliability of the network’s decision making through quantitative evaluation is critical in clinical studies. Therefore, interpretable AI, which can visually represent how and why a CNN makes certain decisions at a human-understandable level, is an essential part of clinical applications. Most recent studies have implemented a class activation map (CAM) to provide visual explanations of CNN output by representing a heatmap that can highlight the decision-relevant regions on the input image.

Our study aims to systematically review published studies that applied deep learning approaches for the diagnosis and prognosis of COVID-19 based on CT and X-ray images. The main contributions of our systematic review are as follows:

We analyzed publicly available CT and X-ray imaging datasets for COVID-19 cases and evaluated their overall performance with conventional and state-of-the-art CNN approaches. We provided a detailed description of CNN architecture in terms of the structural network design and its pros and cons. We examined visualization models that can quantitatively evaluate CNN model decisions. We identified further challenging issues based on our findings of the review to be importantly addressed in the medical AI community to advance the field.

This review is structured as follows. In Section 2, we present the methodology used to carry out the review protocol, search strategy, and data extraction. In Section 3, we provide a summarization of the results in terms of the research perspectives identified in the previous section. Section 4 discusses the limitations of this literature review and suggests future research directions. We believe that this review will aid clinical practice by informing future research and development about improved diagnostic and treatment techniques for patients with COVID-19.

## 2. Methodology

### 2.1. Protocol and Literature Search

The scope of this review included articles reporting the use of convolutional neural network (CNN) methods for chest-computed tomography and X-ray imaging interpretation in COVID-19 patients. We conducted this scoping review in accordance with the PRISMA guidelines [10]: **P**atient population—critical/intensive care patients; **I**ntervention/diagnostic tools—deep learning models; **C**omparison—non-deep learning prediction models; **O**utcomes—prediction of mortality (in ICU); Secondary—the value of deep learning methods in decision making, characteristics of deep learning models, and resources, including data and code availability, AI mode/approach, DL structure, transfer learning, end-to-end learning, explainability, pulmonary involvement (injury) measurement, pulmonary involvement classification, deep learning architectures used in biomedical image analysis, image processing techniques, and the opinions of certified radiologists.

The main goals of this systematic review are: (1) to analyze the original studies and publicly available datasets reporting the application of deep learning in the analysis of COVID-19-related lung involvement, (2) to provide a comprehensive analysis of detection of COVID-19-related pneumonia and segmentation methods based on deep learning, (3) to discuss advantages and disadvantages of the methods and DL methods used (4) to discuss recommendations and future challenges based on the results of the systematic review, and (5) to summarize the limitations of the study and the significance of the application of the deep learning.

Inclusion and exclusion criteria were as follows:

1. The included studies must have assessed the diagnostic or prognostic potential using deep learning algorithms in COVID-19 patients with pulmonary manifestations.
2. Only original studies were included in the systematic review.
3. Abstracts, case reports, case series, invited reviews, narrative and systematic reviews, meta-analyses, animal studies, editorials, letters to the editors, conference papers, commentaries, comparative studies, and expert views were excluded.
4. Articles in non-English language were excluded.
5. Studies that examined non-deep learning applications for diagnosis of pulmonary manifestations in COVID-19 patients were also excluded;
6. The review study excluded papers that did not present sufficient information on related classification performance metrics.
7. Finally, we also excluded studies focusing on non-radiological methods of diagnosis of pulmonary manifestations in COVID-19 patients, even if deep learning methods were used.

**Figure 1:**
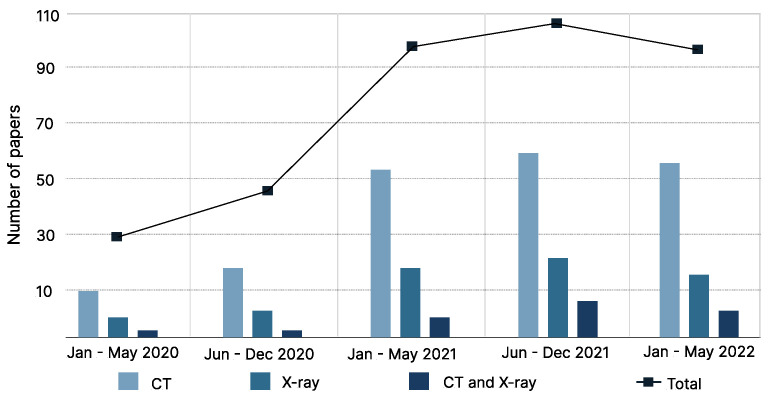
Illustration of meta-analysis based on published papers from January 2020 to May 2022. The systematic review included COVID-19 classification studies based on DNN approach for the CT and chest X-ray imaging dataset.

**Figure 2:**
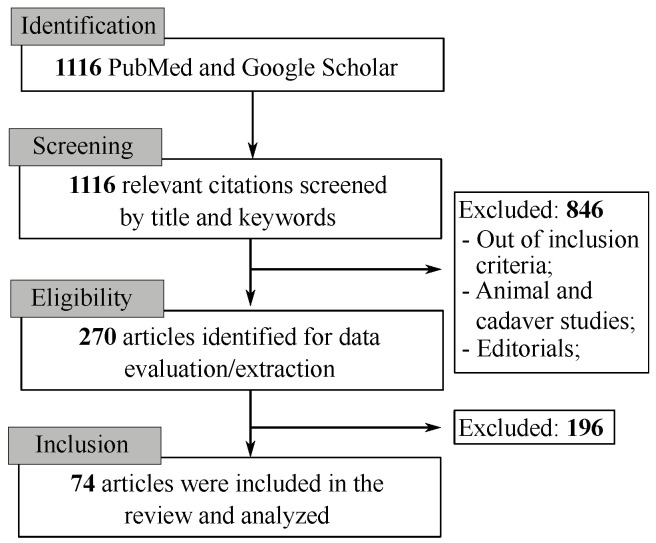
PRISMA flowchart for systematic review and meta-Analysis with the keywords used in the literature review.

The literature search resulted in 1116 citations. Following the removal of duplicates and screening of titles with abstracts, as well as reading the full articles, we analyzed 74 articles in the final version of our systematic review (as depicted in Figure 1 and Figure 2).

The screening of the potential studies was conducted by three reviewers (DV, AS, and MHL). After removal of duplicated titles, we screened the titles and abstracts. The data extraction process was conducted by two reviewers (AS and ML). The discrepancies were resolved by consensus.

We performed a literature search in the following databases: PubMed, EMBASE, and Google Scholar. We used the following free text and medical subject headings (MeSH) terms: ‘lung’, ‘respiratory system’, ‘pneumonia’, ‘respiratory complications’, ‘classification’, ‘artificial intelligence’, and ‘tomography’. The terms were combined with the following words: ‘convolutional neural network’, ‘CNN’, ‘deep learning’, ‘COVID-19’, ‘pneumonia’, ‘detection’, and ‘diagnosis’. We considered all original studies that have been published from inception to May 2022. Articles that did not meet the review criteria were excluded. We examined and cross-referenced bibliographies to identify additional papers of relevance.

In our systematic review, we covered a wide range of attributes related to CT/CXR datasets and DNN approaches. The DNN approaches can be categorized into two directions: image segmentation and classification. Segmentation of chest CXR/CT images involves the automatic localization of boundaries that limit the area of pulmonary involvement (pathological part of the lung), which is then separated from the intact part of the image for further analysis. Image classification entails the ability to capture pixel-level features that may not be easily detected by human eyes. This includes feature representation, pixel-level heatmap visualization, and validation of decoding accuracy.

### 2.2. Specification of Public Dataset

The growth of the deep learning approach has been further facilitated by the creation of large, annotated open datasets. In our review, we focused on deep learning classification models for COVID-19 based on available CT and X-ray scan datasets. We conducted a literature review of papers that used both publicly available open datasets and private datasets. From dataset descriptions, we extracted information on their origin, data types, sample size, image resolutions, populations, available links, and the highest performance achieved in the current research with their CNN approach. Patient scans and other materials included in datasets were typically collected from various sources, such as public domains, hospitals, and physicians, with the collection and distribution of patient information regulated by the ethics review board in each of the reviewed studies. Authors in the reviewed studies reported the use of annotated datasets in which each slice of the CT or X-ray scan was labeled with various classes, including binary classification of COVID-19 pneumonia/normal, COVID-19 pneumonia/bacterial pneumonia, as well as studies reporting multi-class classifications, including three cases of COVID-19, normal, and bacterial pneumonia. For studies reporting the use of public datasets for COVID-19 classification, information on RT-CPR confirmed COVID-19, or radiologist-verified inclusion criteria were checked.

The sample size of datasets varied significantly across different studies. Generally, models fit on smaller datasets showed poor generalization compared to large datasets. Nevertheless, some models were able to achieve high accuracy even on small/moderate-sized datasets.

### 2.3. Performance Evaluation and Baseline Models

Performance evaluation metrics, such as accuracy, sensitivity, specificity, positive predicted value (PPV), negative predicted value (NPV), the area under the curve (AUC), and F-score were extracted. Individual papers presented several baseline models for comparing the performance with the actual proposed model. For review purposes, these evaluation results were recorded for the best-performed baseline models. In some studies, authors only reported a subset of the presented metrics. For some of the studies that reported the performance metrics, we were able to recover the missing value of the metric with the aid of the reported confusion matrix. In the reviewed papers, we additionally gathered information on the evaluation strategy. The evaluation strategy included the subdivision of the dataset into training, validation, and testing subsets. We looked at the percentage proportion of the dataset splits. Another approach that we focused on during the review was selecting information about the cross-validation approach to evaluate the performance. These approaches most commonly included 5-fold and 10-fold cross-validation procedures.

The actual review study excluded the papers in which an insufficient amount of information on related classification performance metrics was presented. Among other factors, we have not included, in the review, studies that only presented segmentation results in their studies.

### 2.4. CNN Architecture

Our systematic review covers various aspects related to neural network structures, including the CNN model, end-to-end learning, explainability, and transfer learning. We examined the features of different CNN architectures, such as layerwise components, parameter size, preprocessing algorithms, optimization techniques, and so on. Feature representation methods helped us to gain insight into the manifold space by transforming the non-linear and high-dimensional feature space into a subspace dimension where we could visually observe the feature distribution for each class. In this context, explainable models were essential to consider, as they allowed us to intuitively understand the CNN decision by highlighting the contribution score on the input medical image at the pixel level.

Other crucial aspects of our survey were transfer learning and fine-tuning approaches. Medical image data have high resolutions, but small sample sizes compared to the computer vision domain, which could lead to overfitting or generalization problems. Transfer learning has been reported to have a significant impact on knowledge transfer across domains, as it allows for the sharing of equivalent convolution filters and the optimal setting of initial parameters in the network, thus resulting in superior performance.

Therefore, our study includes relevant information regarding model explainability and knowledge transfer perspective as well. The results of the included studies are summarized in Table 1 (see Figure 1). Based on the reviewed papers, we compiled a comprehensive table that summarizes the key details of the most notable approaches published before 2022. The table includes the following information, which we believe represents the main contributions of the reviewed studies:

- CNN architecture, i.e., the main underlying baseline model.
- Imaging modality, i.e., CT scan or X-ray.
- The prediction classes, i.e., COVID-19, normal, and pneumonia.
- Pre-processing steps, i.e., data augmentation, image processing methods, feature extraction, and image segmentation.
- Explainability, i.e., the activation heatmaps and grad-CAM visualization.
- Availability of code.
- Performance evaluation metrics, including accuracy, sensitivity, specificity, F-score, AUC, and others.
- Use of transfer learning techniques.
- Specific details of the datasets used.

The reviewed articles included a wide range of network architectures, with specific descriptions of models and their fine-tuning parameters. The most widely used models were ResNet-50, DenseNet-161, VGG-16, ConvLSTM, 3D ResNet-50, InceptionV3, and DenseNet-201. These models were presented as backbone models or as modified architectures with transformations to the model architecture or to the high-level optimization hyperparameters.

Deep learning is often considered a black box, because it is difficult to understand how a neural network arrives at a certain classification choice. To gain insight into the underlying features that contribute to the decision-making of neural networks, the authors of the reviewed papers analyzed the output of the neural networks using visualization methods that mapped regions of the input images. These methods allowed the prediction score of each pixel in the image to be depicted, thus showing how it contributed to the classification decision. The authors relied on techniques such as grad-CAM, AM, RISE, and OS.

## 3. Results

### 3.1. Definition of the Classification Target Classes

The presented models exhibited variability in target class definitions. The majority of publications used two or three target classes, with COVID-19 (C) and non-COVID-19 (N) classes being the most commonly used. Some studies opted for four classes, thereby aiming to distinguish between smaller subcategories of diseases. For instance, in their work [38], the authors used a transfer learning approach based on the ResNet architecture to classify chest X-ray images into four categories, namely, COVID-19 (C), non-COVID-19 (N), bacterial pneumonia (BP), and viral pneumonia (VP). Some studies focused on pneumonia cases and classified scan images into COVID-19 pneumonia (CP) versus non-COVID-19 pneumonia (nCP).

### 3.2. Overall Performance

The main tasks used to train CNN models included detecting, classifying, and segmenting chest CT and chest X-ray images of COVID-19 patients. Most studies defined the classification problem as binary decoding, i.e., COVID-19 (C) vs. non-COVID-19 (N), and also used more specific disease definitions such as bacterial pneumonia (BP), viral pneumonia (VP), COVID-19 pneumonia (CP), non-COVID-19 pneumonia (nCP), COVID-19 stage (ML), medium COVID-19 stage (MD), severe stage of COVID-19 (SC), and non-COVID-19 stage (N).

In our review, the evaluation factor performances ranged from 0.57 to 1.0 for sensitivity, 0.58 to 0.99 for specificity, 0.55 to 0.99 for positive predictive value (PPV), 0.67 to 1.0 for area under the curve (AUC), and 0.7 to 1.0 for the F-score. There was inconsistency in using the assessment phases, with most authors reporting using the training phase followed by validation and testing. Others reported training and validation or training and testing only. The proportion and volume of data used for each of these phases also varied widely.

Several authors reported high performance. For instance, Serte et al. [59] developed a ResNet-50 deep learning model to detect COVID-19 in 3D chest CT scan images and compared it with other deep learning models. The proposed ResNet-50 outperformed all other models with a sensitivity of 100%, specificity of 0.98% (0.95), and AUC value of 96% for detecting COVID-19. Sharifrazi et al. [60] proposed a COVID-19 detection model using X-ray images that were fed to a CNN deep learning model followed by ten-fold cross-validation by an SVM classifier. The proposed CNN-SVM model with a Sobel filter (CNN-SVM + Sobel) achieved the highest classification sensitivity of 100%, specificity of 95.23%, and accuracy of 99.02%.

### 3.3. Evaluation of DNN Architectures

Interestingly, only 20% of CNN studies implemented an end-to-end learning framework, with many studies instead suggesting the use of diverse machine learning techniques on raw imaging data, such as data augmentation (DA), feature extraction (FE), segmentation (SG), or image processing (IP). Table 1 displays reported CNN architectures that have demonstrated superior performance on certain datasets, including CNN and its various modifications (Inception V3, Xception, ResNeXt, CFRCF, 2D-CNN, 3D-CNN, ResNet-50, VGG-16, FBSED, EfficientNetB0-3, Inception_resnet_v2, Xception convolutional auto-encoder neural network, DenseCapsNet (DenseNet + CapsNet), MWSR, CTnet-10, AlexNet, CTnet-10, and CovidAid_V2).

In general, the authors reported improved classification accuracy by combining models with advanced pre-processing methods, using an ensemble of classifiers, or employing advanced fine-tuning to the model architecture. We can categorize the contributions that led to superior performance in the reviewed studies into the following types: (1) Preprocessing and data augmentation, (2) Transfer learning and fine-tuning, (3) Ensemble learning, etc.

Several studies combined two or more approaches in their works. For instance, in [61], the authors used an ensemble of 15 pre-trained models (EfficientNets(B0-B5), NasNetLarge, NasNetMobile, InceptionV3, ResNet-50, SeResnet 50, Xception, DenseNet121, ResNext50, and Inception_resnet_v2) in conjunction with data augmentation (DA) for the binary classification task of COVID-19 on a relatively small dataset.

### 3.4. Ensemble Learning

The purpose of an ensemble system is to correct errors made by other classifiers within the system, and, thus, the diversity of the classifiers is crucial [62]. If all classifiers give the same output, the ensemble system would provide no additional benefit. Therefore, it is essential that the individual classifiers within an ensemble system make different errors in different instances to reduce the total error by accounting for the different errors of each classifier in the ensemble.

Several studies suggested and applied the ensemble learning approach to improve classification accuracy [61,63]. This approach involves combining multiple distinct CNN models to provide more reliable results. The results reported by these studies indicate that the ensemble approach is highly successful in solving the COVID-19 classification problem. One example of the success of the ensemble approach is that it can learn complex non-linear boundaries by combining the classification results of several linear models. For instance, one study used a stacked ensemble of CNN classifiers consisting of ResNet50, ResNet101, VGG16, VGG19, Xception, MobileNetV1, MobileNetV2, DenseNet121, and DenseNet169 models and achieved 99.31% accuracy. In another study, the authors utilized a different ensemble approach that calculated the fuzzy membership values of InceptionV3, DenseNet121, and VGG19 classifiers.

### 3.5. Transfer Learning and Data Augmentation

There have been numerous DNN models used for COVID-19 detection, including pre-trained and novel architectures. The former and the latter are the two most common approaches for addressing the issue. When pre-trained features need to be preserved, transfer learning is used by freezing the convolution part (in the case of a CNN) and unfreezing the dense or classification part of the architecture. Several well-known CNN architectures, such as ResNet, Inception, VGG, AlexNet, DenseNet, ConvNet, DarkNet, 3D-CNN, and LSTM, have been adapted using transfer learning for COVID-19 detection (see Table 1) [64]. On the other hand, fine-tuning is used by freezing the classification part of the architecture and unfreezing the convolution part of the model. Models such as AlexNet, GoogleNet, and SqueezeNet were fine-tuned for COVID-19 detection [65].

The second most common approach is creating a new model specifically for COVID-19 recognition. Models such as CSEn, PF-BAT FKNN, NASNet, DeepSense, DeCoVNet, and ConvLSTM were proposed for the problem. Additionally, popular architectures such as Xception with auto-encoder, DenseCapsNet (DenseNet + CapsNet), DWS-CNN+Deep SVM, VGG-16+nCOV-NET, DenseNet-161+nCOV, ResNet-50+nCOV-NET, UNet+SCOAT, 27-layer 3D-CNN, MobileNetV2+FKNN, and MobileNetV2+FKNN were modified for COVID-19 detection (see Table 1). However, according to Pham [65], the fine-tuned models, such as AlexNet, GoogleNet, and SqueezeNet, outperformed new models proposed for COVID-19 detection, such as CoroNet, CovidGAN, and DarkCovidNet.

Many studies employed additional pre-processing steps before feeding data into deep CNN models, which typically provided better classification results. Data augmentation (DA) methods were used in nearly half of the surveyed studies, with most reporting high accuracy scores after their application. For example, authors [14] reported a 99% accuracy for the ternary classification task (non-COVID-19, COVID-19, pneumonia) using ResNet-V2 architecture by applying a set of augmentations based on the random segmentation of original scans to 480 × 480 pixels, followed by random horizontal flips and normalization. Additionally, many studies employed different approaches for lung segmentation, such as the “snake” technique used by Saad et al. [66], which involves segmenting the lung section to detach the pixels of interest from noise and other unnecessary areas.

### 3.6. Explainability of CNN Model

To add transparency to the proposed models, the majority of reviewed papers implemented visualization techniques. The gradient-weighted class activation mapping (grad-CAM) [67] approach has been the most commonly used method for model explainability. This approach utilizes the gradient information received by the last convolutional layer in a CNN to comprehend the importance of each neuron during the decision-making process [67].

In [12], the authors utilized the occlusion sensitivity (OS) method for visualization purposes. According to the authors, this method provides a clear illustration of the lung regions that are the most crucial for COVID-19 prediction. They concluded that the occlusion sensitivity visualization method they used provided evidence of significant lung regions clearly within the lungs, thereby avoiding sensitivity on corners or edges.

In [13], studies showed the effectiveness of RISE visualization when applied to X-ray images. The results indicated that the RISE technique successfully emphasizes the changes in lung X-ray images caused by COVID-19 infection.

Multiple studies in this survey paper used the activation map visualization technique. In [15], the authors used a spatial activation map which, as they suggested, successfully identified key areas relevant to infected regions in their proposed DA-CMIL model.

In [23], activation maps were also used to illustrate the infected lung regions. To demonstrate the effectiveness of the method, they compared the images obtained using an activation map with the RALE score of those X-ray images. The RALE score represents the severity of the disease. The authors concluded that the visual representation of infected regions obtained by the activation map matched perfectly with the RALE score that had been calculated.

Several papers reviewed did not provide a detailed description of the specific explainable model, and, thus, their approaches were marked as a heat map (HM). In 46% of the papers, the visualization results were provided by highlighting the pixel-level confidence score on the 2D-CXR or 3D-CT image. The heat maps in these studies showed convincing results, as the intra-zone and middle-zone of the pulmonary region have the greatest influence on the decision process.

### 3.7. Performance Enhancement: Novel CNN Strategy

The heterogeneity of the dataset plays a crucial role in the classification performance of state-of-the-art DNN models, as evidenced by the CC-CCII dataset [68]. The highest accuracy achieved on this dataset was 93% by a certain CNN model [41], which is good, but not the best result, for the same task compared to DNN models on other large datasets. Our survey suggests that the differences in classifier performances are related to model structure and resource availability.

To overcome the limitations of unimodal CNN architecture and achieve general performance on diverse datasets, ensemble learning has been proposed. Ensemble learning combines multiple distinct CNN models to represent complex non-linear features and robust decision boundaries. The goal is to reduce total error by strategically combining the model outputs, which is similar to the low pass filtering of noise. For example, one study proposed a stacked ensemble of CNN classifiers consisting of ResNet50, VGG16, Xception, MobileNetV1, and DenseNet169, while other studies used a different ensemble approach by calculating the fuzzy membership values from InceptionV3, DenseNet121, and VGG19 models. Ensemble learning approaches have achieved superior performance compared to a sole CNN model.

Recent studies have proposed various CNN approaches to boost performance by combining several classification strategies and machine learning techniques. For instance, [69] suggested transfer learning and parameter optimization to simultaneously classify X-ray and CT images in a hierarchical architecture, and [61] proposed transferring an ensemble of 15 pre-trained models with data augmentation techniques.

Gupta et al. [70] claimed the effectiveness of CNN models and proposed COVID-WideNet, which is a capsule network with 20 times fewer trainable parameters that are computationally less expensive while preserving model performance. Feature optimization, channel boosting, and recurrent units such as RNN or LSTM have also reported enhanced performance in studies by [71] (Advanced Squirrel Search Optimization Algorithm), [72] (CB-STM-RENet), and [73] (Gated Recurrent Unit).

### 3.8. Open COVID19 Dataset

Radiological images such as chest X-ray (CXR) and chest computed tomography (CT) are often captured at different resolutions, with some of the most common being 512 × 512, 580 × 335, 333 × 308, 299 × 299, 1024 × 1024, 333 × 308, 1766 × 1349, 224 × 224, and 1857 × 1317. These images were obtained from various sources, including intrinsic hospital data sets such as the Wuhan Pulmonary Hospital (China) [37], Honghu and Nanchang hospitals (China) [48], National China Hospitals [68], municipal hospitals in Moscow (Russia) [74], Sao Paulo hospitals (Brazil) [75], the Metropolitan Hospital of Lapa (Brazil) [76], the Indiana University Hospital (USA) [77], and radiology hospitals (Italy) [78].

Additionally, the study utilized open datasets such as the “China Consortium of Chest CT Image Investigation (CC-CCII) Dataset” [68], MosMedData [74], COVID-CT [79], SARS-COV-2 CT Scan [75], Harvard Dataverse [76], LIDC-IDRI [80], iCTCF [81], three versions of COVIDx-CT [47,82,83], COVID-19-CXR [84], COVID-19 RD [85], ChestX-ray8 [86], CXR (COVID-19 and pneumonia) [84,87,88], CT [89], RSNA PDC [86], COVID-19 IDC [88], CDGC [90], and SIRM [78]. Population information, gender ratio, sample size, classes, resolution, and links to public datasets were extracted from the descriptions of these datasets, as are summarized in Table 2.

The majority of datasets and codes are readily accessible and available (see Table 1 and Table 2). Among the surveyed datasets of CT and X-ray scans, the largest in terms of sample size and patient populations were CC-CCII (with 411529 CT slices), CT (with 120968 chest X-ray images), iCTCF (with 256356 CT slices), LIDC-IDRI (with 244527 CT slices), COVIDNet-CT (with 194922 CT slices), COVIDx-CT (with 201103 CT slices), and ChestX-ray8 (with 112120 chest X-ray images).

A certain proportion of these datasets included a combined collection of images from several open-source COVID-19-related or other pulmonary conditions datasets. For example, the CT dataset consisted of multi-source chest X-ray data from the BIMCV, COVID-19 RD, and RSNA PDC datasets. Other large-scale dataset collections were mostly a product of multi-hospital efforts to provide an appropriate and urgent solution to analyze the rapidly growing scan collections within a certain country or region. For instance, the CC-CCII (China Consortium of Chest CT Image Investigation) dataset included patient CT scans from CC-Sun Yat-sen Memorial Hospital, the Third Affiliated Hospital of Sun Yat-sen University, the First Affiliated Hospital of Anhui Medical University, the West China Hospital, the Nanjing Renmin Hospital, the Yichang Central People’s Hospital, and the Renmin Hospital of Wuhan University in China.

New variants of the SARS-CoV-2 virus have emerged in addition to the original strain causing COVID-19. The most prevalent ones are the delta variant, which emerged in late 2020, and the omicron variant, which emerged in late 2022. While all currently available datasets provide CT and CXR images of typical COVID-19 disease, there is a dearth of datasets featuring its variants. In one study [97], an open-access dataset featuring two classes of data—CT scans [98] and X-rays [99]—of the delta and omicron variants was used. For the initial phase of their framework, the researchers used an X-ray database obtained from Kaggle [100,101], along with a limited local database to test it. They also collected a comprehensive database of CT scan images from the radiology centers of Tehran University Hospitals to train and test the model’s second phase, thereby making it entirely native. The definitive status of the cases in this dataset was determined after conducting PCR tests. Another dataset featuring CT scan images of children with delta variant cases is available upon contacting the corresponding author of the paper [102], but it is not open-access. In their study, the authors analyzed delta variant cases in children without using CNN classification.

### 3.9. Review of the Validity and Applicability of the DL Models

The majority of studies across all specialties were deemed high risk according to the ’Quality Assessment of Diagnostic Accuracies Studies 2’ (QUADAS-2) due to significant deficiencies in patient selection, flow, and timing. The PROBAST assessment tool [103] also revealed that all included studies had a high risk of bias, thereby indicating that the model’s performance in practice may be lower than reported. Most studies did not provide specific details about patients and interventions, thus resulting in a high-risk rating for the participant domain.

## 4. Discussion and Conclusions

From the original articles analyzed in this systematic review, automated AI-aided radiological image interpretation has become increasingly useful in highly active and low-resource clinical settings. The reviewed models demonstrated high performance and reliability in detecting pulmonary manifestations of COVID-19 and their differentiations from non-COVID-19 pneumonia.

This study aimed to (1) estimate the diagnostic or/and predictive performance of DL algorithms to identify distinct radiological features of the pulmonary manifestation of COVID-19 using chest CT and chest X-ray images; and to (2) review the variation in the study reporting DL in radiological diagnosis in the published studies. We found that DL algorithms demonstrate a high diagnostic and predictive performance and are acceptable to be used in clinical settings. The high diagnostic and predictive accuracy of DL approaches was identified in all articles, thus suggesting that DL algorithms can be deployed and used for assistance in overloaded clinical settings.

DL models can provide valuable support to doctors in making an accurate diagnosis and facilitate heavy workloads, especially when the healthcare system is overloaded or in resource-constrained regions with a shortage of radiologists [104]. However, the diagnostic accuracy of DL is not significantly higher compared with experienced radiologists. The time spent per CT image interpretation and description was 10 s per scan, while experienced radiologists spent about 10 min for the same task [104].

One study showed that it was feasible to rapidly develop reliable lung segmentation for COVID-19 using deep CNN. Satisfactory results were achieved with less than 50 cases used for training. Deep CNN was successful in developing the fully automated quantification of pulmonary manifestations of COVID-19. The authors trained the deep CNN-based segmentation algorithm and implemented a threshold-based quantification assessing lung opacity load into the clinical practice [105].

One research group tested the performance of 17 neural networks including AlexNet, ResNet-50, DarkNet-53, DarkNet-19, SqueezNet, GoogleNet, Place365-GoogLeNet, MobileNet-v2, ShuffleNet, NasNet-Mobile, Xception, Inception-ResNet-v28, Inception-v3, DenseNet-201, ResNet-18, VGG-19, and ResNet-101. The authors reported that DarkNet-19 outperformed other neural networks in the interpretation of chest X-ray imaging of COVID-19 patients. DarkNet-19 achieved an accuracy value of 94.28% on 5854 X-ray images [106].

The next research group developed a multiple-instance learning method (based on a deep learning method) to accurately predict the disease severity of COVID-19 using quantitative CT data. This model was efficient in identifying patients at high risk for progression in the early phase of COVID-19, which was useful in preventing disease progression and decreasing mortality. The authors recommended that COVID-19 patients undergo CT screening after admittance to the hospital to identify patients at high risk before disease progression [48].

COVIDNet-CT is a deep CNN architecture customized for the detection of pulmonary injury in COVID-19 patients using chest CT images through machine-driven design. The authors created COVIDx-CT, a CT image dataset containing 104,009 images from 1489 patients [47]. Nevertheless, because of high heterogeneity across studies, there was substantial uncertainty of the diagnostic accuracy estimates.

Although CT scanning has shown high performance in the diagnosis of COVID-19, chest X-ray also has many benefits. Thus, it is an easily available approach that can assist radiologists in emergency clinical settings and those widely used worldwide throughout the pandemic. The model has been reported to diagnose COVID-19 for several seconds. Despite the high performance of CT, it is expensive and not widely accessible in low-resource settings. Moreover, the dose of radiation is higher in CT.

Chest X-rays can enable the rapid triaging of patients with pulmonary manifestation and can be performed simultaneously with other laboratory tests to assist medical staff in identifying COVID-19 patients among the large numbers of patients [107]. Existing evidence showed that the chest X-ray plays a fundamental role in the diagnosis of COVID-19. Although the radiological features of pulmonary manifestations in COVID-19 patients and non-COVID-19 pneumonia may have some similarities, key radiological patterns allow clinicians to differentiate between these two diseases [107].

Chest X-rays patterns in COVID-19 patients include (1) ground-glass opacities (usually bilateral, multifocal, peripheral subpleural, medial, basal, and posterior location), (2) a crazy paving appearance (ground-glass opacities with inter- or/and intra-lobular septal thickening), (3) traction bronchiectasis, (4) air space consolidation, and (5) bronchovascular thickening (in the lesion). Chest X-ray features of pneumonia include (1) ground-glass opacities (however, they are opposite to COVID-19 central distribution and unilateral), (2) distribution more along the bronchovascular bundle, (3) vascular thickening, (4) reticular opacity, and (5) bronchial wall thickening. A wide variety of models and solutions have been proposed. One of the proposed models was completely automated and did not require manual feature extraction. The reported accuracy was 98.08% for binary and 87.02% for multi-class tasks [107]. The best and the most commonly used framework for automated classification and detection of the pulmonary manifestations of COVID-19 using chest CT and chest X-ray is the detection and classification CNN.

Chest X-rays are mostly used for detection, while CT scans are mostly used for classification tasks. The segmentation of radiological patterns is primarily executed using CT scans [108]. Transfer learning has been used to accelerate model learning and diminish the requirement for large training data sets by using pre-trained CNN models [108]. Sekeroglu et al. demonstrated that a CNN with minimized fully connected convolutional layers was capable of identifying COVID-19 within two classes with mean ROC AUC scores of higher than 96.0% [56].

Dansana et al. also achieved high performance of the models by using the least computationally intensive deep learning architecture models to detect COVID-19 on chest X-ray images. The VGG-16 model achieved the highest precision of 100% [57]. The next group developed and assessed an AI model using a large dataset including more than ten thousand CT volumes from patients with different pulmonary pathologies (COVID-19, non-COVID-19 viral pneumonia, such as influenza-A/B, non-viral community-acquired pneumonia, and non-pneumonia). This deep CNN model achieved an area under the curve on two publicly available datasets of 92.99% on CC-CCII and 93.25% on MosMedData. The AI model outperformed radiologists [109].

Heidari et al. developed a transfer deep-learning-based CNN model to classify pulmonary involvement in COVID-19 patients using chest X-ray images. The image preprocessing generated better input image data for developing deep learning models. The authors achieved high classification performance, which can be further optimized to detect COVID-19 cases and validated using large and diverse image datasets [110].

The next research group used a decision fusion approach combining the predictions of each of the individual deep CNN models to improve the predictive performance. Such an approach achieved an F1-Score of 0.853 and ROC AUC of 0.824 and reduced false positives. The performance of this model could be further boosted by applying image augmentation transfer learning and feature level fusion [111].

Afshar et al. developed and evaluated a capsule network framework for the diagnosis of COVID-19 using X-ray images. The framework consisted of several capsules and convolutional layers. The model achieved excellent performance with a low number of used parameters. The pre-training process improved the accuracy, specificity, and AUC. The model achieved a sensitivity of 90%, a specificity of 95.8%, an accuracy of 95.7%, and an area under the curve of 0.97. The model is publically available [112].

Jin et al. developed an AI system consisting of five main networks: (1) pulmonary segmentation, (2) slice diagnosis, (3) COVID-infectious slice location, (4) visualization, and (5) image phenotype analysis [109]. The system showed excellent performance on the test sets, with an AUC of 0.9745 for COVID-19, 0.9804 for community-acquired pneumonia, 0.9885 for influenza, and 0.9752 for non-pneumonia. Moreover, for diagnosing COVID-19, the sensitivity was 0.8703, the specificity was 0.9660, and the multi-way AUC was 0.9781. The authors also performed radiomics 36 feature extraction from the attentional regions and identified 665-dimensional imaging features to find the twelve most discriminative features to differentiate COVID-19 from other types of pneumonia. However, there was no significant difference in radiomics features in differentiating influenza from COVID-19 [109].

Although hypoxemic COVID-19 patients share the same etiologic factor (SARS-CoV-2), for severely hypoxemic patients, despite sharing a single etiology (SARS-CoV-2), their pulmonary manifestations can be quite different from one another. The spectrum of respiratory failure can vary from normal breathing (“silent” hypoxemia) to markedly dyspneic, as well as from responsive to certain treatments, such as nitric oxide or prone positioning, to not. Therefore, even the same disease can be characterized by heterogeneous clinical manifestation. The same disease actually presents itself with impressive difference. Two phenotypes (L and H) were proposed, which are best differentiated by CT. Therefore, artificial intelligence can potentially be useful in phenotype identification and can assist in understanding pathophysiology, which is crucial to establish appropriate treatment [113]. Even though PCR is being used globally for COVID-19 diagnostics and tracking, CCT processed by CNN showed much better diagnostic accuracy. Thus, Lacerda et al. developed a CNN model that achieved a sensitivity of 97%, precision of 82%, and an accuracy of 88%, thereby outperforming the diagnostic accuracy of human experts (72%) based on CCT interpretation and the sensitivity of the PCR tests (53–88%) [28].

### 4.1. Limitations of CNN in COVID-19 Detection

A majority of studies did not perform an external validation of the algorithm on a separate test dataset and used results from the internal validation data. This might have led to an overestimation of the diagnostic performance. The overfitting was well described across many studies [111].

Although some studies reported high model performance values, they had been validated (tested) using the intrinsic database, whereas the true performance value of the algorithms can be ultimately concluded after the use of external validation (test) on separate external test datasets with previously unused data from representatives of the target population. Another limitation was an imbalance between the study groups, as well as a small sample size of the severe patients included in the study [114].

CNN models require large datasets that have all possible variants of data for achieving the highest accuracy. The shortage of available datasets is a barrier to their training. A majority of models have been trained and validated on small datasets [108]. To counteract the inefficiency of the training datasets and increase their size, several authors reported using GAN models for data augmentation. Such data augmentation made the model more robust to overfitting [108]. Another limitation includes the so-called “black box”, which is the basic feature of deep neural networks. Even though attention maps assist in interpretation by highlighting the dominant areas, they are not fully sufficient to visualize the unique features used by CNNs to differentiate between COVID-19 and non-COVID-19 pneumonia. Since the majority of studies primarily focused on radiological diagnostics (chest CT and X-ray), clinical information was not included [108].

There was also an extensive variation in the terminology, study methodology, data interpretability, and outcome. It was very difficult to formally assess the performance of algorithms due to the variation in reporting. The variability in study methodology and reporting can be partially explained by the fact that these studies were conducted by researchers from different specialties, including medical specialists (intensivists, emergency physicians, pulmonologists, radiologists), information technology specialists, and engineers. Therefore, the study goals and the experience of researchers also varied from study to study. Even though DL algorithms showed high diagnostic accuracy in medical imaging, it is, at present, difficult to determine if they are fully applicable in real clinical settings.

### 4.2. Potential for Future Research

The presence of large image datasets is necessary to improve generalizability and limit overfitting in training CNNs. Although techniques have improved learning on small datasets, large medical datasets are essential. The majority of achievements of CNNs are mainly based on a large amount of data.

Therefore, creating large datasets of radiological images is one of the main challenges that must be addressed by the research community of clinicians and computer scientists, as well as hospital administrators and other staff involved in AI research. Although building large clinical datasets is expensive and requires enormous work by specialists, an international collaborative effort of the medical network is necessary to advance this field. Such collaborative work should follow common guidelines for the systematic acquirement and annotation of radiological images without interfering with the clinical routine [115].

Medical datasets should ideally be annotated by certified radiologists, and this process is very time-consuming. Applications such as GTCreator [116] can contribute to ground truth creation and facilitate image annotations, sharing, and revising conclusions among different radiologists [115]. It is generally recommended to establish a bootstrapping methodology for testing sets, indicating the number of images, which images should be considered in each iteration, and the sample size and repetitions [115]. Further improvement of the diagnostic performance of the DL models could be achieved of including clinical and laboratory data.

Building large clinical datasets is expensive and requires enormous work by specialists. It is an essential procedure in the DNN approach for model reliability. The international collaborative effort of the medical network is necessary to advance this field. A more effective method is to use a generative adversarial network for generating new CT/CXR images that can contribute to the continuation of the network learning.

While numerous models have been developed, the inter-exchange of data between the sources would result in achieving better performance. Moreover, the customization of these models according to the local patient population using regionally collected data would also improve the models’ performance. Finally, the most important task for the future would be shifting from research-based model development and testing to actual implementation and continuation of testing such models in the real-world environment.

Although deep learning is currently one of the fastest developing fields in medicine with the potential to improve the diagnosis of pulmonary manifestations in COVID-19 patients, some conclusions about its superiority over clinicians seem to be over-promising. Moreover, since the beginning of the pandemic, hundreds of studies have been conducted; however, the evidence on the implementation of deep learning models into real-world clinical settings and the assessment if their value is still missing. Almost all studies reported excellent performance, and some of them even outperformed certified radiologists in making image-based diagnoses. However, many studies either did not describe exactly how they assessed DL models against doctors, or the process of assessment was not rigorous enough to support the conclusions. One study reported the opinions of radiologists about the DL models, but these opinions were rather subjective and not standardized. Despite there being a massive explosion of research focusing on DL in the diagnosis of COVID-19, there is not enough evidence of the actual deployment of these models in clinical practice. A few questions should be addressed before these models get actively used in clinical practice:

1. Can these models actually take over radiologist, or should their usage be strictly limited to a doctor’s assistantship, second opinions, or double-checking?
2. How deeply can healthcare workers rely on these models depending on the level of hospital and doctor availability? Can these models be used without the radiologist’s opinion or only in the case that the radiologist is absolutely unavailable? Or, in this case, should a radiologist definitely double-check the model-made diagnosis?
3. Can these models be used in the region where a particular model has not been validated or accustomed?

Therefore, the conduction of high-quality studies with transparent reporting of the results is necessary to avoid hype, assist healthcare workers, and reduce or avoid harm to patients. Future research should be focused on post-deployment, region-specific validation, and safety in clinical settings. The additional potential pathway that should be considered for achieving advancements in the field is solving patients’ privacy and ethical requirements.

## Figures and Tables

**Table 1:** List of selected publications for CNN classification of CT/X-ray scans.

Publication		Dataset			CNN Architecture					Performance Evaluation			
Year	Study	Source	Imaging	Classes	End-to-End	Expl	Structure	TL	Code	Sens/Spec/PPV/NPV	AUC	*F*_1_	Acc.
2021	[11]	QaTa-Cov19	CXR	4, BP, VP, N, C	no/DA	no	CSEN	yes	no	0.98/0.95/0.64/0.99	-	0.77	0.95
2021	[12]	SARS CoV-2 CT	CT	2, C, N	no/DA	OS	PF-BAT FKNN	yes	no	0.99/0.99/0.99/0.99	0.99	0.99	0.99
2021	[13]	AIIMS	CXR	2, C, N	yes	RISE	CovidAid_V2	no	yes	0.88/0.94/0.88/0.94	-	0.88	0.92
2021	[14]	COVIDx CT-2A	CT	3, C, P, N	no/DA	CAM	ResNet-v2	yes	no	0.98/0.99/0.98/0.99	-	-	0.99
2021	[13]	Shahid Beheshti University of MS	CT	2, C, N	no/FE	no	NASNet	no	no	0.99/0.98/0.99/0.99	-	0.99	0.99
2021	[15]	Yeungnam University	CT	2, P, C	no/DA	AM	DA-CMIL	no	no	1/0.97/0.96/1	0.98	0.98	0.98
2021	[16]	COVIDx	CXR	3, C, P, N	no/DA	no	RCoNet	no	no	0.97/0.98/0.97/-	-	0.97	0.97
2021	[17]	COVID-19 IDL, RSNA, CXR DI	CXR	3, C, P, N	no/DA	CAM	ResNet50	yes	no	0.92/0.97/0.98/-	0.98	-	0.94
2021	[18]	Guangzhou, Hebei	CT	2, C, N	no/DA	no	ResUNet	no	no	0.91/0.90/0.89/0.92	0.90	-	0.91
2021	[19]	Covid-ct-dataset, Guangxi Univ.	CT	2, C, N	no/IP	HM	ResNet50	yes	no	0.93/0.92/-/-	0.93	0.92	0.93
2021	[20]	NLMMC	CT	2, C, N	no/DA	no	GARCD	yes	no	0.967/0.912/-/-	0.98	-	-
2021	[21]	Kaggle Chest X-ray	CXR	2,C, N	no/DA	no	COVINet	no	no	0.98/0.96/0.98/-	0.98	0.98	0.97
2021	[22]	6 Public datasets	CT, CXR	2, C, N	yes	CAM	MDA-BN	no	no	0.98/0.92/0.93/0.98	0.98	0.95	0.95
2021	[23]	COVIDx, 5 US and 4 SK hospitals	CXR	3, C, P, N	no/SG	AM	DL CBIR	no	no	0.85/-/0.95/-	0.83	-	0.83
2021	[24]	4 CH, 2 GH, China	CT	2, C, N	no/IP	no	COVIDNet	no	yes	0.93/0.95/0.93/0.94	0.98	0.93	0.94
2021	[25]	Jihan Infectious Disease Hospital	CT	2, C, P	no/FE	no	DL-MLP	no	no	0.87/0.90/0.80/-	0.92	0.84	0.89
2021	[26]	the Second Xiangya Hospital	CT	2, SC, nSC	no/CR	AM	MIL	no	no	0.93/0.96/-/-	0.98	0.89	0.95
2021	[27]	COVID chest X-ray, Chest X-ray14	CXR	2, C, CP	no/IP & DA	CAM	DenseNet-161	yes	no	0.80/1/0.8/0.96	-	0.98	0.97
2021	[28]	MosMed, LUNA16	CT	2, C, N	no/IP	CAM	VGG16	yes	no	0.97/0.82/0.79/0.97	-	0.89	0.88
2021	[29]	COVID-chest X-ray, CoronaHack COVID-19 radiography	CXR	3, N, P, C	no/IP	CAM	DenseCapsNet	no	no	1.00/0.95/0.91/1.00	-	0.91	0.91
		COVID-CT	CT							0.94/0.99/0.99/0.96	0.96	0.98	0.98
2021	[30]	Covid Chest X-ray	CXR	2, C, N	no/DA	no	ConvLSTM	no	no	0.97/0.98/0.98/0.98	0.81	0.98	0.95
2021	[31]	COVID-CT	CT	2, C, N	no/IP	no	DNN	no	no	0.94/0.96/0.96/0.95	-	0.95	0.95
2021	[32]	COVID-19 RD	CXR	2, C, N	no/DA	no	CheXNet	yes	no	0.99/1.00/1.00/0.99	-	0.99	0.99
		SIRM, COVID-19 X-ray	CXR							0.92/0.97/0.89/0.96	-	0.90	0.93
2021	[33]	COVID-CT, Radiopedia	CT	3, N, C, P	no/DA	HM	EATC	no	no	0.86/0.97/0.90/0.89	-	0.88	0.87
2021	[34]	Covid Chest Xray	CXR	2, C, N	no/FE	no	DWS-CNN	no	no	0.98/0.98/-/-	-	0.98	0.98
2021	[35]	Multi-center dataset	CT	2, C, N	yes	CAM	3D-CNN	yes	yes	0.90/0.98/0.96/0.94	0.88	0.93	0.88
2021	[36]	UCSD (California)	CXR	3, N, C, P	no/IP	no	ANN	no	no	1.00/0.98/0.96/1.00	0.77	0.98	0.94
2021	[37]	Wuhan Pulmonary Hospital	CT	2, SC, nSC	yes	AM	3D ResNet	yes	yes	0.86/0.88/-/-	0.92	-	0.88
2021	[38]	Guangzhou W&C MC	CXR	4, N, C, BP, VP	no	no	Res-CovNet	yes	no	0.97/-/0.97/-	-	0.98	0.98
		SARS-COV-2	CT							0.98/0.98/0.98/0.99	0.98	0.98	0.98
2021	[39]	Harvard Dataverse	CT	2, C, N	yes	no	VGG-11+Inceptionv3+ WideResnet-50	yes	yes	0.99/0.99/.0.98/0.98	0.98	0.98	0.98
2021	[40]	Kaggle Chest X-ray	CXR	3, N, P, C	no	no	HOG+CNN	no	no	0.96/0.99/0.94/0.99	0.99	0.99	0.96
		CC-CCII Dataset	CT							0.90/0.90/-/-	0.89	-	0.93
2021	[41]	COVID-19 IDL, HwaMei Hospital	CXR	3, N, P, C	no/IP	AM	CMT-CNN	no	yes	0.92/0.91/-/-	0.92	-	0.97
		COVID-19 IDL, Chest X-ray-NIHCC	CXR							0.99/0.99/0.99/0.99	-	0.99	0.97
2021	[42]	Curated X-ray Dataset	CXR	3, N, C, P	yes	no	COVID-DeepNet	no	no	0.97/0.98/0.97/0.98	0.99	0.99	0.98
2021	[43]	COVID-19 RD, Mendeley	CXR	3, C, VP, N	no	no	Cov19-CNnet	no	no	0.94/0.96/0.98/1.00	-	0.94	0.98
2021	[44]	BIMCV, COVIDx, COVID-CXNet	CXR	3, N, C, P	no/IP	CAM	Fus-ResNet50	no	no	0.95/0.99/0.94/0.99	-	0.95	0.95
2020	[45]	IEEE8023, COVID-CT, CORD-19	CT	2, C, N	no/FE	no	DeepSense	no	no	0.97/0.97/-/-	-	0.92	0.98
2020	[46]	COVID-chestxray, CORD-19	CXR	2, C, P	no/SG & IP	AM	DarkNet-19	no	yes	1.00/0.97/-/-	-	-	0.98
2020	[47]	COVIDx-CT	CT	3, NC, NCP, CP	no/CR	no	COVIDNet-CT	no	no	0.97/0.99/0.99/0.99	-	0.98	0.99
2020	[48]	Honghu and Nanchang hospitals	CT	3, NS, S, DP	no/CR	no	ResNet34	yes	yes	0.98/0.83/0.85/0.97	0.95	0.91	0.90
2020	[49]	LIDC	CT	2, N, C	no/CR	CAM	DenseNet-121	no	yes	0.91/0.93/0.85/0.95	0.94	0.88	0.90
2020	[50]	COVID-19 CXR	CT	3, N, C, P	no/SG & CR	HM	VGG16	yes	yes	0.91/0.93/-/-	0.89	0.91	0.88
2020	[51]	COVID-chest X-ray	CXR	3, C, P, N	no/IP	no	Inception-V3	yes	yes	1.00/0.99/0.99/0.99	-	0.99	0.99
		COVID-chest X-ray	CT							0.81/0.99/0.97/-	0.95	0.88	0.96
2020	[52]	Daegu, South Korea	CT	2, N, C	no/DA & IP	no	Xception	yes	no	0.95/0.88/0.94/0.91	0.92	0.94	0.95
2020	[53]	COVID-19 X-ray	CXR	2, N, C	no/IP	no	OGA-ELM	no	yes	0.97/0.95/0.95/0.97	0.98	0.96	0.96
		Wuhan People’s Hospital	CT							0.94/0.96/-/-	0.98	-	-
2020	[54]	COVIDx	CXR	3, C, P, NonP	no/IP	CAM	COVNet	yes	yes	0.95/0.95/0.90/0.97	-	0.92	0.93
2020	[55]	COVID-19 CT	CT	2, C, N	no/UNet SG	no	DensNet-201	yes	yes	0.87/0.95/-/-	0.97	0.92	0.92
2020	[56]	COVID chest X-ray	CXR	3, C, N, P	no/IP	no	ConvNet	yes	no	0.94/0.99/0.93/0.99	0.96	0.94	0.98
2020	[57]	COVID chest X-ray	CXR	3, N, P, C	no/DA	no	VGG-16	no	no	0.94/0.94/1.00/0.84	-	0.97	0.91
2020	[58]	PACS Union Hospital	CT	2, C, N	yes	CAM	DeCoVNet	no	yes	0.90/0.91/0.84/0.98	0.95	0.87	0.90

**Table 2:** CT/X-ray public datasets and state-of-the-art performance.

Link	Name	Origin	Type	Resolution	N. Patients	Classes	Sample Size	H.Per.	CNN Model
[68]	CC-CCII	National hospitals (China)	CT	512 × 512	2742	3; C, P, N	411,529	0.93	CMT-CNN [41]
[74]	MosMedData	Municipal hospitals in Moscow (Russia)	CT	512 × 512	1110	4; ML, MD, SC, CC, N	-	0.88	ED-VGG16 [28]
[79]	COVID-CT	medRxiv and bioRxiv (USA)	CT	1853 × 1485	216	2; C, N	812	0.98	ConvLSTM [30]
[75]	SARS-COV-2 CT Scan	Sao Paulo hospitals (Brazil)	CT	327 × 307	1252	2; C, N	2481	0.99	PF-BAT FKNN [12]
[76]	Harvard Dataverse	HSPM (Brazil), Metropolitan Hospital of Lapa (Brazil)	CT	-	210	3; C, N, OLI	4173	0.98	VGG-11+Inceptionv3+ WideResnet-50 [39]
[80]	LIDC-IDRI	Combined: NCI (Malaysia), FNIH and FDA (USA)	CT	512 × 512	1010	4; nonP, CAP, Infl, C	244,527	0.90	DenseNet-121 [49]
[47]	COVIDx CT 1	CNCB (China), ITAC (Canada), LIDC-IDRI, Radiopaedia (Australia)	CT	512 × 512	3745	3; N, CP, nonCP	194,922	0.99	ResNet-v2 [14]
[81]	iCTCF	HUST-UH/HUST-LH (China)	CT	512 × 512	1170	3; SC, nSC, N	256,356	0.98	GARCD [20]
[82]	COVIDx-CT	CNCB (China), ITAC (Canada), LIDC-IDRI, Radiopaedia (Australia)	CT	512 × 512	4501	3; N, CP, nonCP	201,103	0.99	ResNet-v2 [14]
[91]	BIMCV-COVID19	Medical Imaging Databank in Valencian Region MIB (Spain)	CT CXR	-	1354	3; UD, N, P	23,527	0.95	MDA-BN [22]
[84]	COVID-19 CXR	BMJ, Doctors Without Borders, Mount Sinai Health System (Canada)	CXR	1594 × 1600	48	3; C, P, N	55	0.96	Xception [52]
[85]	COVID-19 RD	SIRM (Italy), COVID-19 IDC (Canada), PadChest (Spain), RSNA (USA), COVID-CXNet	CXR	299 × 299	-	3; C, N, VP	3615	0.99	CheXNet [32]
[86]	ChestX-ray8	National Institute of Health Clinical Center (USA)	CXR	1024 × 1024	30,805	3; C, P, N	112,120	0.99	COVID-DeepNet [42]
[87]	OCTaCXRI	Guangzhou Women and Children’s Medical Center (China)	CXR	different	-	2; P, N	5856	0.98	Res-CovNet [38]
[92]	Curated X-Ray Dataset	Indian Institute of Science, PES Uni., Ramaiah IT (India), Concordia Uni. (Canada)	CXR	479 × 479	-	4; C, N, VP, BP	9208	0.98	DenseNet [93]
[94] [84,88]	CXR (COVID-19 & Pneumonia)	COVID-19 IDC (Canada), Mendeley (UK), COVID-19 CXR (Canada)	CXR	386 × 386	-	3; C, P, N	6432	0.96	HOG+CNN [40]
[77]	Open-i CXR	Indiana University Hospital (USA)	CXR	-	-	2; CovidP, OP	7470	0.97	DenseNet-161 [27]
[89]	CT	BIMCV (Spain), RSNA PDC (USA), COVID-19 RD (Bangladesh)	CXR	224 × 224	-	4; C, N, VP, BP	120,968	0.95	CSEN [11]
[86]	RSNA PDC	Radiological Society of North America (USA)	CXR	-	11,254	3; N, NLO, LO	26684	0.94	ResNet50 [17]
[83]	COVIDx CT 2	SIRM (Italy), COVID-19 IDC (Canada), Radiopaedia (Australia), COVID-19 CXR (Canada), Hannover Uni. (Germany)	CXR	859 × 730	4501	3; CP, CAP, N	201,103	0.95	Fus-ResNet50 [44]
[84,88] [84,95] [86]	COVIDx	COVID-19 IDC (Canada), MIDRC (USA), Actualmed (Spain), COVID-19 RD, RSNA PDC (USA), COVID-19 CXR (Canada)	CXR	-	15100	2; C, NC	16,000	0.97	RCoNet [16]
[88]	COVID-19 IDC	Radiopaedia (Australia), SIRM (Italy), RSNA PDC (USA), Eurorad (Germany), Coronacases (China), COVID-19 CXR (Canada)	CXR	604 × 499	412	2; C, P	679	0.98	DWS-CNN [34]
[90]	COVID-19 PL CXR I	unreported	CXR	-	-	2; C, N	98	0.97	COVINet [96]
[90]	CDGC	unreported	CXR	-	-	2; VP, BP	79	0.97	COVINet [96]
[78]	SIRM	Radiology Hospitals (Italy)	CXR	356 × 338	115	1; C	450	0.93	DAM [33]

## Data Availability

Not applicable.
